# Sperm DNA integrity and sexual dysfunction among infertile men: DFI and sexual dysfunction

**DOI:** 10.1007/s10815-025-03697-x

**Published:** 2025-10-28

**Authors:** Gilad Karavani, Justin Chan, Yulia Wilk Goldsher, Katherine Lajkosz, Susan Lau, Kirk C. Lo, Ethan D. Grober, Yonah Krakowsky, Keith Jarvi

**Affiliations:** 1 Division of Urology, Department of Surgery, Mount Sinai Hospital, University of Toronto, Toronto, Ontario Canada; 2 Division of Urology, Department of Surgery, University of Toronto, Toronto, ON Canada; 3 Women’s College Hospital, University of Toronto, Toronto, Ontario Canada; 4 Faculty of Medicine, Tel Aviv University, Tel Aviv, Israel; 5 Department of Biostatistics, Princess Margaret Cancer Centre, Toronto, Ontario Canada

**Keywords:** DNA fragmentation, Sexual dysfunction, Intercourse frequency, Androgen deficiency, SHIM score, ADAM score

## Abstract

**Purpose:**

To evaluate the association between sperm DNA fragmentation index (DFI), which quantifies the proportion of sperm with damaged DNA (sperm DNA fragmentation), and sexual dysfunction (SD) using the Sexual Health Inventory for Men (SHIM), a validated 5-item tool assessing erectile dysfunction severity, and the Androgen Deficiency in Aging Male (ADAM) questionnaire, a 10-item screening instrument for symptoms of testosterone deficiency.

**Methods:**

A retrospective cohort study was conducted at a university-affiliated male infertility clinic. A total of 703 infertile men (mean age 37.4 ± 5.6 years) who completed SHIM and ADAM questionnaires and underwent semen analysis and DFI testing between 2000 and 2020 were included. DFI was categorized as normal (< 30%) or abnormal (≥ 30%). Primary outcomes were intercourse frequency (IF), SHIM scores (erectile dysfunction severity), and ADAM scores (androgen deficiency symptoms). Multivariable regression models evaluated predictors of sexual function, with emphasis on DFI.

**Results:**

Abnormal DFI was observed in 39% of men. Average IF was 7.2 ± 4.4 times/month, with no difference by DFI status. A positive ADAM score was reported in 41.1%, while moderate/severe ED (SHIM) was reported in 3%. Multivariable analysis showed that BMI above 30 (kg/m²) alone was associated with reduced IF. Abnormal SHIM scores were predictive of positive ADAM score. Worse SHIM scores were associated with smoking and a positive ADAM score. Men with abnormal DFI had significantly lower SHIM scores (*p* = 0.02): 65% had normal scores versus 73% in the normal DFI group. Mild and mild-moderate ED were reported in 25% and 9% of the abnormal DFI group versus 19% and 5% in the normal group, respectively.

**Conclusion:**

Abnormal DFI was significantly associated with erectile dysfunction. These findings support incorporating sexual health assessments into male infertility evaluations.

## Introduction

Infertility is defined as the failure to achieve a pregnancy after a year or more of regular unprotected sexual intercourse. Globally, it has been estimated that infertility affects 48.5 million couples [1]. While male factor infertility contributes to roughly 50% of infertility, isolated male factor infertility is responsible for 20–30% of all infertility cases [2, 3]. 

In men, infertility can significantly impact sexual function. While sexual dysfunction is a direct cause of infertility in 2.3% of cases, it also influences fertility through both direct and indirect mechanisms [4]. Sexual dysfunction is broadly manifested in four different disorders: sexual desire, arousal, orgasmic, and pain disorders [5].

Male sexual dysfunction is common in the general population, and 20–30% of adult men worldwide report at least one type of sexual dysfunction [5, 6], and prevalence increases with increased age.

There has been limited research showing that SD is more prevalent within the population of men with infertility, including a recent meta-analysis reporting a higher frequency of erectile dysfunction [7–9] and a similar [8, 9] or higher [7] prevalence of premature ejaculation in the men of an infertile couple, compared with what is observed in the general male population of a similar age. But this association and the causative mechanisms are yet to be fully understood.

Erectile dysfunction (ED), an arousal disorder, is one of the most common causes of sexual dysfunction in men and has been found to be more prevalent in infertile men than the general population [6]. While there is limited research investigating the relationship between sexual dysfunction and fertility [10], a recent cross-sectional study by Lotti et al. demonstrated that the incidence of ED was found to increase as a function of semen quality impairment severity [7]. In their study, men diagnosed with azoospermia exhibited the most severe impairments in sexual function and overall health. Psychopathological traits, along with a generally less healthy phenotype, were identified as significant factors contributing to the development of ED.

One semen parameter that is not routinely tested as part of conventional semen analysis is sperm DNA fragmentation (SDF). While the routine inclusion of SDF testing in semen analysis remains debated, growing evidence supports its strong association with reproductive outcomes [9]. High levels of SDF have been linked to male infertility, particularly in the context of in vitro fertilization (IVF), where elevated SDF is correlated with lower fertilization rates, diminished embryo quality, increased pregnancy loss rates, and reduced pregnancy rates [11]. Currently, no studies have directly explored the potential association between male sexual dysfunction and SDF.

We used validated and widely accepted tools for male sexual dysfunction evaluation, including the Sexual Health Inventory for Men (SHIM), a commonly used abridged version of the International Index of Erectile Function that grades erectile dysfunction severity [12], and the Androgen Deficiency in the Aging Male (ADAM) questionnaire, a 10-item screening tool for androgen deficiency symptoms [13]. In addition, we included intercourse frequency (IF) as a behavioral indicator of sexual activity. The objective of this retrospective study is to assess male sexual dysfunction in men with infertility and to evaluate its relationship with sperm DNA integrity. By utilizing the SHIM scores and ADAM assessments with behavioral parameters like IF, we aim to provide a comprehensive evaluation between sexual dysfunction and male infertility.

## Methods

In this study, we assessed a cohort of patients who attended a tertiary male infertility clinic in Ontario, Canada, from 2000 to 2020. This multidisciplinary andrology center, dedicated to male reproductive health, specializes in andrology—including male infertility and male sexual dysfunction. The clinical team consists of urologists with subspecialty training in andrology, supported by andrology laboratory staff and nursing. All patients attending the clinic filled out a comprehensive intake questionnaire covering various aspects: demographics, anthropometrics, medical, surgical, and fertility history, details regarding sexual dysfunction, testosterone deficiency, depressive symptoms, and substance use. Specifically, the questionnaire delved into details such as cigarette consumption (measured in packs per day: none, less than 1, 1, or 2), alcohol intake (ranging from none to up to 4 drinks per week), cannabis usage, and cocaine consumption.

Sexual function was assessed using three complementary measures: IF, reported as the number of sexual encounters per month; the SHIM questionnaire, which evaluates erectile dysfunction severity; and the ADAM questionnaire, which screens for symptoms of androgen deficiency.

Specifically, patients provided information on the frequency of sexual intercourse (per month), and this data was further categorized as 1–3, 4–7, 8–11, and 12 + times per month.

Patients additionally filled out the validated SHIM questionnaire. The SHIM score categories, as previously described by Rosen et al. [12], are “normal,” “mild,” “mild/moderate,” “moderate,” and “severe” reflecting increasing risk for ED. Due to the limited number of patients with a SHIM score classified as “moderate” and “severe,” these scores were merged into a single group named “moderate/severe” (for some comparisons, the category “mild/moderate” was also included in the above category).

Additionally, patients underwent assessment for symptoms of androgen deficiency by completing the ten-question ADAM questionnaire [13]. Patients are considered to have a positive ADAM assessment, indicating potential symptoms of androgen deficiency, if they meet any of the following criteria: answering “yes” to question 1, responding affirmatively to question 7, or indicating “yes” to at least three out of ten questions.

This dataset was integrated with laboratory tests, encompassing hormone panels and semen analyses. All assessments were performed within the same laboratory setting at Mount Sinai Hospital. Hormonal evaluations included follicle-stimulating hormone (FSH, measured in mIU/mL), luteinizing hormone (LH, mIU/mL), estradiol (pmol/L), prolactin (ng/mL), and testosterone (nmol/L).

Participants’ body mass index (BMI) was calculated as weight in kilograms divided by height in meters squared (kg/m^2^). BMI data was collected and categorized according to standard classifications: normal weight (< 24.9 kg/m^2^), overweight (25.0–29.9 kg/m^2^), and obese (≥ 30.0 kg/m^2^). These categories were used for subgroup analyses and comparisons across study groups. Additionally, depressive symptoms were assessed by two validated questions, “During the past month, have you often been bothered by feeling down, depressed, or hopeless?” and “During the past month, have you often been bothered by little interest or pleasure in doing things?” [14]. Scores were quantified by the answers “not at all,” “several days,” “more than half the days,” and “nearly every day,” for both of these questions. If patients reported depressive symptoms, the patients were directly questioned about suicidal thoughts.

Semen samples were obtained following a 2- to 7-day period of abstinence. Analysis of the samples adhered to the 2010 WHO criteria (15), encompassing volume (in milliliters), concentration (in millions per milliliter), motility (expressed as a percentage), normal morphology (as a percentage), and sperm viability (as a percentage). Sperm concentration and motility were determined following the evaluation of duplicate samples from each semen specimen. Evaluation of sperm morphology involved microscopic examination for structural characteristics in accordance with WHO guidelines [15].

The most used parameter to quantify SDF is the DNA fragmentation index (DFI), measured by flow cytometry–based assays. DFI was assessed in our patient cohort by analyzing frozen, prepared semen samples using the sperm chromatin structure assay (SCSA). As previously outlined by Samplaski et al. [16], each sample was first exposed for 30 s to 400 μL of a solution containing 0.1% Triton X-100, 0.15 M NaCl, and 0.08 N HCl (pH 1.2). This was immediately followed by the addition of 1.2 mL of staining buffer (6 μg/mL acridine orange, 37 mM citric acid, 126 mM Na_2_HPO_4_, 1 mM disodium EDTA, and 0.15 M NaCl, pH 6.0). The samples were then analyzed by flow cytometry. Upon excitation with a 488-nm wavelength light source, acridine orange bound to double-stranded DNA fluoresced green (515–530 nm), while acridine orange bound to single-stranded DNA emitted red fluorescence (≥ 630 nm). Flow cytometric analysis was performed three minutes after staining using a FACSCalibur Flow Cytometer (Becton Dickinson, San Jose, California). At least 5000 cells were evaluated per sample, and the proportion of sperm showing abnormal red fluorescence emission, indicative of denatured DNA, was recorded.

All DFI measurements reported in this study were conducted at the same laboratory at Mount Sinai Hospital with a DFI of 30% or more defined as an abnormal DFI level [9].

We included in this study men referred to our male infertility clinic who filled the intake questionnaire, performed semen analysis including DFI tests in our laboratory, and filled the ADAM and SHIM questionnaires as well. Excluded were patients who performed the SA and DFI tests outside of our laboratory as well as the small group of patients not actively trying to conceive.

Based on DFI values, we divided our study into normal and abnormal DFI to compare intercourse frequency (IF), ADAM, and SHIM scores as well as SA data and basic characteristics. We additionally divided our cohort according to IF, ADAM assessment (positive vs. negative) and SHIM severity categories (“normal,” “mild,” “mild-moderate,” and “moderate/severe” and “severe,” as higher SHIM score reflects increasing dysfunction and higher risk for ED) for the purpose of creating regression models to identify predictors for these outcomes. Demographic, past medical and fertility history, sperm parameters and hormone levels, body mass index (BMI) (recorded from 2013 onward), and substance use were compared between groups as well.

### Statistical analysis

Demographic and clinical characteristics were summarized using descriptive statistics. Results were stratified by frequency of sexual intercourse per month (categorized as 1–3, 4–7, 8–11, and 12 +), ADAM score (positive vs. negative), and SHIM score (categorized as normal, mild, moderate, and moderate/severe). Differences in characteristics across groups were assessed using the Mann–Whitney and Kruskal–Wallis tests for continuous characteristics and the chi-squared and Fisher’s exact tests for categorical characteristics.

Regression models using demographic and clinical characteristics to predict the frequency of sexual intercourse (as categorized above), positive ADAM assessment, and SHIM score were fit. Due to the small number of patients with moderate/severe SHIM scores, moderate and moderate/severe were collapsed into a single category for modelling. Ordinal regression models were used to model intercourse frequency and SHIM score, and logistic regression models were used to model positive ADAM assessment. For each outcome, univariable regression models incorporating patient characteristics were fit for each outcome evaluated. Predictors with a significant *p*-value (< 0.05) were included in the multivariable regression models. To improve model fit, characteristics with skewed distributions were logarithmically transformed.

All statistical analyses were performed using the R Foundation for Statistical Computing software, version 4.0. All tests were two-tailed and a *p*-value of < 0.05 was considered statistically significant.

### Ethics

Before completing the intake questionnaire, all participants signed an informed consent form approved by the Institutional Review Board (IRB). Approval from the institutional Research Ethics Board (approval number 14–0342-E) was obtained for both data collection and analysis.

## Results

### Study population

Between January 2000 and January 2020, 3549 men with SA data completed the intake questionnaire, which included the ADAM and SHIM assessments. Among them, 703 underwent DFI testing, forming the final cohort for analysis. The mean age of the cohort was 37.4 ± 5.6 years. Their average testosterone level was 13.8 ± 5.7 (range 1.9–44.0) nmol/L, with average FSH and LH levels of 5.6 ± 3.8 and 5.1 ± 2.5 mIU/mL, respectively.

Among the 700 men (out of 703) with available data on IF, the average frequency of intercourse per month was 7.2 ± 4.4 times. Additionally, 41.1% of the participants recorded a positive ADAM assessment, and 3% reported moderate/severe SHIM scores. The mean DFI was 29.0 ± 17.2%, with 429 men (61%) having a normal DFI (< 30%) and 274 men (39.0%) showing an abnormal DFI (≥ 30%).

### Intercourse frequency

Basic characteristics, age, BMI, alcohol consumption, smoking, depressive symptoms, semen parameters, and hormone levels by IF per month (1–3, 4–7, 8–11, and 12 + times) are summarized in Table 1. Patients in higher intercourse frequency groups were significantly younger (*p* < 0.001). Semen volume and other sperm parameters did not differ between the groups. BMI, depressive symptoms and FSH, LH, prolactin, and testosterone levels were also similar between the IF groups. The mean DFI levels and rates of abnormal DFI were also compared between the IF groups and showed no significant difference (*p* = 0.29 and *p* = 0.22, respectively).

**Table 1:** Comparison of basic characteristics, sperm parameters and hormone levels by frequency of sexual intercourse per month

	1–3 (*n* = 131)	4–7 (*n* = 266)	8–11 (*n* = 195)	12 + (*n* = 108)	*p*-value
Age	39.6 (5.4)	37.5 (5.2)	36.5 (5.9)	36.2 (5.6)	** < 0.001**
BMI (kg/m^2^)					0.59
≤ 24.9	20 (29.4)	28 (26.4)	27 (33.3)	17 (30.4)	
25–29.9	34 (50.0)	52 (49.1)	44 (54.3)	27 (48.2)	
30 +	14 (20.6)	26 (24.5)	10 (12.3)	12 (21.4)	
Missing	63	160	114	52	
Alcohol consumption	72 (55.0)	182 (68.4)	121 (62.1)	61 (56.5)	**0.032**
Smoking	16 (12.2)	34 (12.8)	27 (13.8)	21 (19.4)	0.34
Frequency of feeling down, depressed, or hopeless in the past month					0.81
Not at all	35 (66.0)	50 (60.2)	33 (61.1)	23 (67.6)	
Several days	15 (28.3)	25 (30.1)	17 (31.5)	9 (26.5)	
More than half the days	3 (5.7)	8 (9.6)	3 (5.6)	1 (2.9)	
Nearly every day	0 (0.0)	0 (0.0)	1 (1.9)	1 (2.9)	
Missing	78	183	141	74	
Frequency of little interest or pleasure in doing things in the past month					0.95
Not at all	40 (75.5)	56 (66.7)	39 (73.6)	23 (67.6)	
Several days	11 (20.8)	22 (26.2)	12 (22.6)	9 (26.5)	
More than half the days	2 (3.8)	5 (6.0)	2 (3.8)	1 (2.9)	
Nearly every day	0 (0.0)	1 (1.2)	0 (0.0)	1 (2.9)	
Missing	78	182	142	74	
Sperm volume (ml)	2.8 (1.4)	2.9 (1.5)	2.9 (1.3)	2.7 (1.4)	0.69
Sperm motility (%)	23.9 (12.0)	22.5 (11.0)	22.7 (11.1)	21.0 (11.7)	0.21
Sperm morphology (%)	12.9 (10.2)	12.2 (9.5)	11.3 (8.4)	11.3 (10.0)	0.49
Sperm viability (%)	63.3 (17.3)	65.3 (16.2)	65.5 (16.4)	64.1 (17.8)	0.68
Sperm concentration (M/ml)	46.9 (50.7)	45.3 (48.3)	37.1 (41.1)	34.6 (44.3)	0.065
Sperm DFI (%)	30.9 (17.7)	29.5 (17.3)	28.1 (16.6)	27.7 (17.6)	0.29
DFI category					0.22
< 30%	71 (54.2)	160 (60.2)	123 (63.1)	72 (66.7)	
≥ 30%	60 (45.8)	106 (39.8)	72 (36.9)	36 (33.3)	
Testosterone	12.9 (4.5)	13.8 (6.1)	13.6 (5.7)	15.2 (6.3)	0.077
Missing	50	129	82	43	
FSH	6.6 (5.2)	5.1 (3.2)	5.3 (3.0)	5.8 (4.0)	0.27
Missing	49	127	82	43	
LH	5.4 (2.8)	4.7 (2.3)	5.1 (2.4)	5.3 (2.4)	0.15
Missing	49	127	83	43	
Prolactin	8.5 (4.2)	8.8 (4.6)	8.7 (3.9)	9.5 (5.9)	0.93
Missing	51	115	76	40	

Data are presented as mean (SD), or as *n* (%).*DFI*, DNA fragmentation index; *BMI*, body mass index; *FSH*, follicle-stimulating hormone; *LH*, luteinizing hormone

Bold values represent statistical significancy - a *p*-value <0.05

The relationship between the predictors of interest (age, smoking, BMI, alcohol consumption, sperm parameters, hormones and DFI levels) and IF was modelled using a multivariable ordinal regression model (Suppl. Table 1). This model demonstrated that BMI above 30 (kg/m^2^) (OR 0.43 [95% CI 0.20–0.91], *p* = 0.027), is negatively associated with IF, while age, alcohol, smoking, sperm parameters, and hormone levels are not significantly associated with IF. DFI category (< 30% and ≥ 30%) was not found to be a significant predictor (OR 0.99 [95% CI 0.54–1.84], *p* = 0.98).

### Prediction of positive ADAM

We stratified our cohort by categories of ADAM assessment—positive vs. negative. Basic characteristics, age, BMI, cigarette smoking, alcohol consumption and depressive symptoms as well as sperm parameters, DFI data, and hormone levels were compared between the two ADAM category groups (positive and negative) and presented in Table 2.

**Table 2:** Comparison of basic characteristics, sperm parameters and hormone levels by categorical ADAM questionnaire score results (positive vs. negative)

	Negative ADAM (*n* = 412)	Positive ADAM (*n* = 291)	*p*-value
Age	36.9 (5.1)	38.2 (6.2)	**0.011**
BMI (kg/m^2^)			0.20
≤ 24.9	66 (32.8)	26 (23.2)	
25–29.9	96 (47.8)	61 (54.5)	
30 +	39 (19.4)	25 (22.3)	
Missing	211	179	
Alcohol consumption	261 (63)	177 (61)	0.55
Smoking	54 (13)	44 (15)	0.52
Frequency of feeling down, depressed, or hopeless in the past month			** < 0.001**
Not at all	106 (76.8)	37 (42.0)	
Several days	28 (20.3)	38 (43.2)	
More than half the days	2 (1.4)	13 (14.8)	
Nearly every day	2 (1.4)	0 (0.0)	
Missing	274	203	
Frequency of little interest or pleasure in doing things in the past month			** < 0.001**
Not at all	114 (82.6)	44 (50.0)	
Several days	21 (15.2)	35 (39.8)	
More than half the days	2 (1.4)	8 (9.1)	
Nearly every day	1 (0.7)	1 (1.1)	
Missing	274	203	
Semen volume (ml)	2.9 (1.4)	2.8 (1.5)	0.14
Sperm motility (%)	22.7 (11.1)	22.5 (11.8)	0.72
Sperm morphology (%)	11.2 (9.2)	13.1 (9.7)	**0.008**
Sperm viability (%)	65.6 (15.7)	63.9 (18.0)	0.43
Sperm concentration (M/ml)	39.7 (44.0)	44.8 (49.5)	0.22
DFI (%)	28.2 (15.7)	30.2 (19.1)	0.60
DFI category			0.34
< 30%	258 (63)	171 (59)	
≥ 30%	154 (37)	120 (41)	
Testosterone	14.4 (6.0)	12.9 (5.1)	**0.013**
Missing	168	137	
FSH	5.4 (4.0)	5.8 (3.5)	**0.034**
Missing	166	136	
LH	5.0 (2.5)	5.1 (2.4)	0.57
Missing	167	136	
Prolactin	8.5 (4.2)	9.3 (5.0)	0.11
Missing	158	125	
SHIM score category			** < 0.001**
Normal	343 (83)	147 (51)	
Mild	57 (14)	91 (31)	
Moderate	9 (2)	35 (12)	
Moderate/severe	3 (1)	18 (6)	

Data are presented as mean (SD), or as *n* (%).*DFI*, DNA fragmentation index; *BMI*, body mass index; *FSH*, follicle-stimulating hormone; *LH*, luteinizing hormone; *ADAM*, The Androgen Deficiency in Aging Males; *SHIM*, The Sexual Health Inventory for Men

Bold values represent statistical significancy - a *p*-value <0.05

Patients with positive ADAM assessment were older (38.2 ± 6.2 vs. 36.9 ± 5.1 years, *p* = 0.011) and reported a higher occurrence of depressive symptoms—feeling down, depressed, or hopeless, and little interest or pleasure in activities during the past month (*p* < 0.001 for both questions). The positive ADAM group also had lower levels of testosterone (12.9 ± 5.1 vs. 14.4 ± 6.0 nmol/L, *p* = 0.013), higher FSH levels (5.8 ± 3.5 vs. 5.4 ± 4.0 mIU/mL, *p* = 0.034), and higher rates of normal morphology sperm (13.1 ± 9.7% vs. 11.2 ± 9.2%, *p* = 0.008). SHIM score also differed between the ADAM groups, with higher percentage in each abnormal SHIM score category (*p* < 0.001) (Table 2).

A multivariable logistic regression model was fit to test the relationship between age, BMI, smoking status, alcohol consumption, semen analysis data, hormone levels, DFI levels and SHIM score (categorical), and the dependent parameter of positive ADAM assessment (Table 3). The abnormal SHIM score categories mild and moderate/severe (compared to the normal category) were found to be significantly predictive of a positive ADAM assessment (Table 3). Age and testosterone were not independently associated with a positive ADAM score.

**Table 3:** Multivariable logistic regression model for prediction of positive ADAM questionnaire score

	OR (95%CI)	*p*-value
Age	1.027 (0.970, 1.088)	0.36
BMI (kg/m^2^)		
≤ 24.9	Reference	
25–29.9	1.997 (0.883, 4.518)	0.097
30 +	1.724 (0.632, 4.700)	0.29
Smoking		0.85
No	Reference	
Yes	0.906 (0.328, 2.502)	
Alcohol		0.73
No	Reference	
Yes	1.136 (0.555, 2.327)	
Log semen volume	0.985 (0.417, 2.325)	0.97
Sperm motility	1.005 (0.961, 1.051)	0.83
Log sperm morphology	1.318 (0.856, 2.030)	0.21
Log sperm concentration	0.974 (0.664, 1.426)	0.89
Sperm viability	0.989 (0.959, 1.020)	0.50
DFI category		0.58
< 30%	Reference	
≥ 30%	0.786 (0.337, 1.832)	
SHIM		
Normal	Reference	
Mild	3.926 (1.768, 8.718)	** < 0.001**
Moderate/severe	11.632 (3.804, 35.564)	** < 0.001**
Testosterone	0.961 (0.901, 1.025)	0.23
Log FSH	1.241 (0.491, 3.135)	0.65
Log LH	2.350 (0.686, 8.044)	0.17
Log prolactin	1.935 (0.883, 4.242)	0.099

*DFI*, DNA fragmentation index; *BMI*, body mass index; *FSH*, follicle-stimulating hormone; *LH*, luteinizing hormone; *ADAM*, The Androgen Deficiency in Aging Males; *SHIM*, The Sexual Health Inventory for Men

Bold values represent statistical significancy - a *p*-value <0.05

### SHIM severity score prediction

Basic characteristics, age, BMI, alcohol consumption, smoking status, depressive symptoms, and semen analysis data including DFI levels and hormone levels were stratified by SHIM score—"normal,” “mild,” and “moderate/severe”—and presented in Table 4. The mean age gradually increased with SHIM score severity (36.9 ± 5.3 in the “normal” group compared to 38.2 ± 6.4 and 39.2 ± 5.2in the “moderate/severe” groups, respectively, *p* < 0.001). Alcohol consumption decreased with SHIM score category severity (66% vs. 58% and 42%, respectively; p < 0.001).

**Table 4:** Comparison of basic characteristics, sperm parameters and hormone levels by SHIM questionnaire score category

	Normal (*n* = 490)	Mild (*n* = 148)	Moderate/ severe (*n* = 65)	*p*-value
Age	36.9 (5.3)	38.2 (6.4)	39.2 (5.2)	** < 0.001**
BMI (kg/m^2^)				0.050
≤ 24.9	60 (29.0)	24 (33.3)	8 (23.5)	
25–29.9	105 (50.7)	39 (54.2)	13 (38.2)	
30 +	42 (20.3)	9 (12.5)	13 (38.2)	
Missing	283	76	31	
Alcohol consumption	325 (66.3)	86 (58.1)	27 (41.5)	** < 0.001**
Smoking	61 (12.4)	25 (16.9)	12 (18.5)	0.21
Frequency of feeling down, depressed, or hopeless in the past month				** < 0.001**
Not at all	105 (72.4)	26 (46.4)	12 (48.0)	
Several days	34 (23.4)	24 (42.9)	8 (32.0)	
More than half the days	4 (2.8)	6 (10.7)	5 (20.0)	
Nearly every day	2 (1.4)	0 (0.0)	0 (0.0)	
Missing	345	92	40	
Frequency of little interest or pleasure in doing things in the past month				**0.004**
Not at all	113 (77.9)	34 (60.7)	11 (44.0)	
Several days	27 (18.6)	18 (32.1)	11 (44.0)	
More than half the days	4 (2.8)	3 (5.4)	3 (12.0)	
Nearly every day	1 (0.7)	1 (1.8)	0 (0.0)	
Missing	345	92	40	
Semen volume (ml)	2.8 (1.4)	2.8 (1.5)	2.8 (1.5)	0.61
Sperm motility (%)	22.9 (11.1)	21.8 (11.6)	22.6 (13.1)	0.60
Sperm morphology (%)	11.5 (9.1)	12.5 (10.0)	13.8 (10.8)	0.26
Sperm viability (%)	66.3 (15.2)	61.8 (18.7)	61.1 (20.9)	**0.029**
Sperm concentration (M/ml)	40.4 (43.5)	43.8 (50.8)	47.7 (56.1)	0.71
DFI (%)	27.5 (15.0)	32.9 (20.7)	32.0 (21.8)	0.087
DFI category				0.092
< 30%	312 (63.7)	81 (54.7)	36 (55.4)	
≥ 30%	178 (36.3)	67 (45.3)	29 (44.6)	
Testosterone	14.3 (6.0)	13.1 (5.5)	12.4 (4.1)	0.10
Missing	220	66	19	
FSH	5.5 (3.7)	5.7 (4.4)	5.5 (3.2)	0.90
Missing	219	65	18	
LH	5.1 (2.5)	4.7 (2.5)	5.5 (2.4)	0.077
Missing	220	65	18	
Prolactin	8.7 (4.7)	9.0 (4.4)	8.9 (4.2)	0.64
Missing	205	58	20	
ADAM positive				** < 0.001**
Negative (%)	343 (70.0)	57 (38.5)	12 (18.5)	
Positive (%)	147 (30.0)	91 (61.5)	53 (81.5)	

Data are presented as mean (SD), or as *n* (%).*DFI*, DNA fragmentation index; *BMI*, body mass index; *FSH*, follicle-stimulating hormone; *LH*, luteinizing hormone; *ADAM*, The Androgen Deficiency in Aging Males; *SHIM*, The Sexual Health Inventory for Men

Bold values represent statistical significancy - a *p*-value <0.05

A progressively higher occurrence of depressive symptoms—feeling down, depressed, or hopeless, and little interest or pleasure in activities during the past month (*p* < 0.001 and *p* = 0.004, respectively)—was observed with increasing SHIM severity. Most semen parameters as well as DFI (continuous and by categories (< 30% vs. ≥ 30)) were similarly comparable in the SHIM score groups, except for sperm viability, which was decreased in the “mild” and “moderate/severe” groups, compared to the “normal” SHIM score group (62% and 61% vs. 66%, respectively, *p* = 0.029). FSH, LH, prolactin, and testosterone levels were also similar in the SHIM score groups (Table 4). However, a positive ADAM score was more commonly observed in individuals with higher severity SHIM score categories (30% in the “normal” group, 62% in the “mild” group, and 82% in the “moderate/severe” group, *p* < 0.001).

A multivariable ordinal regression model was used to assess the relationships between several predictors, including age, BMI, smoking habits, alcohol consumption, sperm parameters, DFI categories, hormone levels, and SHIM score categories. The SHIM scores were categorized as “normal,” “mild,” “mild-moderate,” and “moderate/severe,” with increasingly abnormal SHIM scores reflecting a higher risk of ED (Table 5). This model was able to show that a positive ADAM assessment (OR 5.81 [95% CI 2.94–11.48], *p* < 0.001) and smoking (OR 3.05 [95% CI 1.23–7.57], *p* = 0.016) were positively and independently associated with increasingly abnormal SHIM score.

**Table 5:** Multivariable logistic regression model for prediction SHIM questionnaire score

	OR (95%CI)	*p*-value
Age	1.02 (0.97, 1.08)	0.49
BMI (kg/m^2^)		
≤ 24.9	Reference	
25–29.9	0.57 (0.26, 1.25)	0.16
30 +	1.54 (0.62, 3.80)	0.35
Smoking		**0.016**
No	Reference	
Yes	3.05 (1.23, 7.57)	
Alcohol		0.085
No	Reference	
Yes	0.55 (0.28, 1.09)	
Log semen volume	0.94 (0.42, 2.10)	0.89
Sperm motility	1.00 (0.96, 1.04)	0.93
Log sperm morphology	1.06 (0.70, 1.59)	0.80
Log sperm concentration	1.29 (0.89, 1.85)	0.17
Sperm viability	0.99 (0.96, 1.02)	0.61
DFI category		0.17
< 30%	Reference	
≥ 30%	1.77 (0.78, 4.03)	
ADAM positive		** < 0.001**
Negative	Reference	
Positive	5.81 (2.94, 11.48)	
Testosterone	0.96 (0.90, 1.02)	0.17
Log FSH	0.95 (0.39, 2.30)	0.90
Log LH	1.89 (0.58, 6.08)	0.29
Log Prolactin	0.79 (0.37, 1.71)	0.56

*DFI*, DNA fragmentation index; *BMI*, body mass index; *FSH*, follicle-stimulating hormone; *LH*, luteinizing hormone; *SHIM*, The Sexual Health Inventory for Men; *ADAM*, The Androgen Deficiency in Aging Males

Bold values represent statistical significancy - a *p*-value <0.05

### Abnormal DFI levels and sexual dysfunction evaluation

We compared the sexual function related parameters between patients according to their categorical DFI levels—normal (< 30%) vs. abnormal (≥ 30%) (Table 6). The normal DFI group (*n* = 429) was similar to the abnormal DFI group (*n* = 274) in terms of IF per month (7.4 ± 4.4 vs. 6.8 ± 4.3 times, *p* = 0.06) and per category (0, 1–3, 4–7, 8–11, 12 + , *p* = 0.20) and showed similar mean ADAM score (1.7 ± 2.1 vs. 1.8 ± 2.2, *p* = 0.83) and categorical positive score (40% vs. 44%, *p* = 0.34). However, SHIM score results did differ between the groups, as the abnormal DFI group presented with lower rates of “normal” score (65% vs. 73%) and higher rates of scores “mild” and “moderate” (25% vs. 19% and 9% vs. 5%, respectively, *p* = 0.02). BMI and depressive symptoms were comparable between the DFI groups.

**Table 6:** Comparison of sexual function related parameters according to DNA fragmentation index (normal vs. abnormal results)

	DFI < 30% (*n* = 429)	DFI ≥ 30% (*n* = 274)	*p*-value
BMI (kg/m^2^)	52 (29.5)	40 (29.2)	0.31
≤ 24.9	83 (47.2)	74 (54.0)	
25–29.9	41 (23.3)	23 (16.8)	
30 +	253	137	
Missing	52 (29.5)	40 (29.2)	
Frequency of feeling down, depressed, or hopeless in the past month			0.74
Not at all	86 (65.6)	57 (60.0)	
Several days	37 (28.2)	29 (30.5)	
More than half the days	7 (5.3)	8 (8.4)	
Nearly every day	1 (0.8)	1 (1.1)	
Missing	298	179	
Frequency of little interest or pleasure in doing things in the past month			0.84
Not at all	91 (68.9)	67 (71.3)	
Several days	35 (26.5)	21 (22.3)	
More than half the days	5 (3.8)	5 (5.3)	
Nearly every day	1 (0.8)	1 (1.1)	
Missing	297	180	
Sexual intercourse frequency (per month)	7.4 (4.4)	6.8 (4.3)	0.060
Sexual Intercourse Category			0.20
0	3 (0.7)	0 (0.0)	
1–3	71 (16.6)	60 (21.9)	
4–7	160 (37.3)	106 (38.7)	
8–11	123 (28.7)	72 (26.3)	
12 +	72 (16.8)	36 (13.1)	
ADAM-Q score sum			0.83
Mean (sd)	1.7 (2.1)	1.8 (2.2)	
Median (Q1,Q3)	1 (0, 3)	1 (0, 3)	
Range (min, max)	(0, 9)	(0, 9)	
Positive ADAM score			0.34
Negative	258 (60.1)	154 (56.2)	
Positive	171 (39.9)	120 (43.8)	
SHIM score categories			**0.015**
Normal	312 (72.7)	178 (65.0)	
Mild	81 (18.9)	67 (24.5)	
Moderate	20 (4.7)	24 (8.8)	
Moderate/Severe	16 (3.7)	5 (1.8)	

Data are presented as mean (SD), or as *n* (%). Note: *BMI*, body mass index; *SHIM*, The Sexual Health Inventory for Men; *ADAM*, The Androgen Deficiency in Aging Males

Bold values represent statistical significancy - a *p*-value <0.05

## Discussion

In this study, we evaluated correlations between male fertility variables including sperm parameters and DNA fragmentation and sexual dysfunction. Sexual dysfunction was assessed using the SHIM questionnaire (erectile dysfunction severity), the ADAM questionnaire (androgen deficiency symptoms), and intercourse frequency as a behavioral measure of sexual activity. In this cohort, IF was evaluated categorically with results showing that BMI > 30 correlates with less frequent intercourse sessions, while all other parameters were not associated with IF in the regression model.

In models analyzing predictors of ED based on SHIM scores, smoking and positive ADAM assessment were independently linked to a higher risk of severe SHIM scores. The bidirectional relation between the sexual dysfunction parameters was also evident when the analysis of abnormal SHIM scores was positively associated with the prediction of positive ADAM assessments.

While the validity of the SHIM score for SD and specifically ED has been previously investigated, the relation between abnormal SHIM score and ADAM assessment is less explored. The strong association seen in our cohort emphasizes the value of combining SD evaluation.

The results regarding sperm variables in this cohort are interesting, especially when evaluating the clinical aspect of DFI. While 39% of the cohort showed abnormal DFI levels (≥ 30%), no significant difference in IF and ADAM scores between men with normal and abnormal DFI levels was observed. The abnormal DFI group demonstrated more severe sexual dysfunction, with higher rates of “mild” and “moderate” scores. Specifically, men with abnormal DFI were less likely to have normal SHIM scores and more likely to present with “mild” and “moderate” sexual dysfunction.

Beyond the physiological impact of impaired DFI, the psychological awareness of poor semen quality may independently contribute to sexual dysfunction. Men who are informed of abnormal semen parameters—particularly in the emotionally charged context of infertility—may experience increased performance anxiety, reduced sexual confidence, and heightened psychological distress, all of which are known contributors to erectile dysfunction [8, 17, 18]. This feedback loop between infertility-related stress and sexual function may further exacerbate the clinical presentation, emphasizing the value of addressing both psychological well-being and medical factors when managing male infertility. In this cohort, depression was associated with positive ADAM scores and abnormal SHIM scores, as evidenced by a significantly higher prevalence of depressive symptoms—such as feeling down, depressed, or hopeless, and experiencing little interest or pleasure in activities—within these groups. However, due to missing data, this variable was not included in the regression models to preserve the statistical power of the analysis.

Several studies have explored the association between sperm quality and sexual dysfunction, using sexual dysfunction validated tools. One important study by Lotti et al. [7] investigated the relationship between the severity of infertility and sexual dysfunction in males of infertile couples, comparing their outcomes to a control group of healthy, fertile men of similar age. The study demonstrated that impaired semen quality is associated with a higher prevalence of ED, as measured by the SHIM score—an abridged, validated version of the International Index of Erectile Function-15 (IIEF-15) [19]—thereby establishing a link between the severity of semen quality impairment and the prevalence of ED.

Although limited research has been conducted on the relationship between SDF and sexual dysfunction, some studies have examined this link alongside other sperm parameters. Yu et al. [20] investigated the incidence of male sexual dysfunction in couples who experienced pregnancy loss, focusing on men with varying degrees of semen quality impairment. One of the groups was comprised of 87 men with abnormally high SDF. The authors found that these men had a significantly higher risk of ED, as measured by the IIEF-5, compared to normozoospermic men. A similar study, which evaluated 437 men of couples with pregnancy loss, confirmed a significant correlation between IIEF-5 scores and sperm parameters. The authors showed that men with elevated IIEF-5 scores had poorer sperm progressive motility, abnormal morphology, and SDF levels [21]. However, although an association between sperm quality and elevated SDF and sexual dysfunction was observed, the underlying physiological mechanisms remain unclear. To the best of our knowledge, no study has established a causal link or elucidated biological pathways connecting sperm DNA damage and sexual dysfunction. Future research, including mechanistic and animal studies, is needed to better understand potential pathways linking the two. Moreover, while these studies emphasize the connection between SDF and sexual dysfunction, underscoring the importance of assessing SDF in infertile men, their findings, restricted to couples experiencing pregnancy loss, may not be generalizable to the broader male infertility population. In contrast, our study focuses on a more generalized population of infertile men, providing a broader perspective on the relationship between SDF and sexual dysfunction.

Previous studies support the idea that androgen deficiency, particularly low testosterone levels, is associated with poor sperm quality [22, 23]. Wang et al. discussed how limitations in standard semen analysis often overlook the hormonal aspects that can influence sperm quality, such as testosterone deficiency, which plays a critical role in spermatogenesis, leading to decreased sperm concentration and quality and thus impact male fertility [23].

Androgen deficiency is widely recognized as a key contributor to ED. A recent review [24] comprehensively examined the mechanisms through which low androgen levels impair erectile function, highlighting oxidative stress (OS) as a significant pathway of damage. One of these mechanisms is by inducing OS, which negatively affects the corpus cavernosum cells [24] by reducing endothelial function and nitric oxide bioavailability, thereby worsening erectile function. Additionally, several studies have demonstrated that OS causes damage to sperm DNA integrity, leading to an increase in SDF [9, 25]. This shared mechanism of oxidative stress could provide a possible explanation for the observed link between elevated DFI and an increased risk of ED, as reflected by higher SHIM scores in patients with elevated DFI.

In this study, although testosterone levels were found to be associated with IF (in the multivariable ordinal regression model) and ADAM score (lower in the negative ADAM score), no significant correlation was observed in regression models between testosterone levels and sexual dysfunction, as reflected by the severity of the SHIM score and ADAM abnormalities in the regression models. This lack of a direct association may be attributed to the multifactorial mechanisms that influence sexual dysfunction in this infertile cohort, as opposed to the general population. This emphasizes that having a laboratory normal range of testosterone levels is insufficient for assuming sexual function, and the SD evaluation should include direct questionnaires to evaluate for ED, IF, and androgen deficiency symptoms.

As previously shown, SD is highly prevalent in the male infertility population, contributing to higher psychological stress and overall adverse quality of life [26]. The exact cause-effect mechanisms are yet to be fully understood, but our study reveals multiple influences within this dysfunction, specifically depressive symptoms, when fertility is compromised. This strengthens the role of sexual function assessment in a comprehensive approach to treating men with infertility.

One of the key strengths of this paper lies in the comprehensive use of both the SHIM and ADAM questionnaires to evaluate male sexual dysfunction and its association with SDF. This dual approach provides a broader perspective, integrating both physiological and psychological (ED) and hormonal (androgen deficiency) aspects of male infertility, enhancing the study’s clinical relevance. Additionally, the large sample size and long follow-up period (20 years) contribute to the robustness of the findings, allowing for a thorough analysis of trends in sperm quality and sexual function.

However, the retrospective design presents some inherent weaknesses. The reliance on existing medical records limits the ability to control for all confounding variables, such as psychological or relationship factors that may influence sexual function but were not consistently recorded. A possible limitation of this study is the lack of metabolic parameters, such as blood glucose or diabetes status, which are relevant to sexual health. Nevertheless, related factors including BMI, depression evaluation, alcohol use, and smoking status were assessed and provided meaningful context. Future studies should consider including comprehensive metabolic profiling to further explore their impact on sexual function. Furthermore, selection bias may be present as the study population is derived from a specialized infertility clinic, which could affect the generalizability of the findings to the broader male population.

## Conclusions

In this large, retrospective study of infertile men, we found that elevated SDF (DFI ≥ 30%) was associated with worse SHIM scores, suggesting a possible link between impaired SDF and erectile dysfunction. However, no significant differences were observed in IF or ADAM scores between men with normal and abnormal DFI, indicating that the relationship between DFI and sexual dysfunction may be limited or multifactorial. In contrast, multivariable regression models revealed more robust associations—smoking and a positive ADAM assessment were independently associated with more severe erectile dysfunction, while SHIM severity was a strong predictor of ADAM positivity. Testosterone levels were negatively correlated with ADAM positivity, though not directly linked to SHIM score severity.

These findings underscore the value of integrated sexual health assessment—including both SHIM and ADAM tools—when evaluating infertile men. Given the potential impact of modifiable factors such as age, smoking, and alcohol use on both sexual function and sperm quality, a comprehensive, multidisciplinary approach is warranted. Prospective studies are needed to further elucidate the mechanistic links between sexual dysfunction and sperm DNA integrity.

## Data Availability

Full dataset is available from the corresponding author, upon reasonable request.

## References

[1] Mascarenhas MN, Flaxman SR, Boerma T, Vanderpoel S, Stevens GA. National, regional, and global trends in infertility prevalence since 1990: a systematic analysis of 277 health surveys. PLoS Med. 2012;9:e1001356. 23271957 10.1371/journal.pmed.1001356 PMC3525527

[2] Agarwal A, Mulgund A, Hamada A, Chyatte MR. A unique view on male infertility around the globe. Reprod Biol Endocrinol. 2015;13:37. 25928197 10.1186/s12958-015-0032-1 PMC4424520

[3] Brugh VM 3rd, Lipshultz LI. Male factor infertility: evaluation and management. Med Clin North Am. 2004;88:367–85. 15049583 10.1016/S0025-7125(03)00150-0

[4] Lotti F, Maggi M. Sexual dysfunction and male infertility. Nat Rev Urol. 2018;15:287–307. 29532805 10.1038/nrurol.2018.20

[5] Berger MH, Messore M, Pastuszak AW, Ramasamy R. Association between infertility and sexual dysfunction in men and women. Sex Med Rev. 2016;4:353–65. 27872029 10.1016/j.sxmr.2016.05.002

[6] Lotti F, Corona G, Rastrelli G, Forti G, Jannini EA, Maggi M. Clinical correlates of erectile dysfunction and premature ejaculation in men with couple infertility. J Sex Med. 2012;9:2698–707. 22897716 10.1111/j.1743-6109.2012.02872.x

[7] Lotti F, Corona G, Castellini G, et al. Semen quality impairment is associated with sexual dysfunction according to its severity. Hum Reprod. 2016;31:2668–80. 27733531 10.1093/humrep/dew246

[8] Yu X, Zhang S, Chen L, Zhang XY, Wang Q. High incidence of sexual dysfunction and timed intercourse was found only in infertile males who have known impairment of sperm quality for a long period: evidence from a hospital-based cross-sectional study. Reprod Biol Endocrinol. 2022;20:139. 36114509 10.1186/s12958-022-01010-4 PMC9479282

[9] Agarwal A, Majzoub A, Baskaran S, Panner Selvam MK, Cho CL, Henkel R. Sperm DNA fragmentation: a new guideline for clinicians. World J Mens Health. 2020;38:412–71. 32777871 10.5534/wjmh.200128 PMC7502318

[10] Liu Y, Wang Y, Pu Z, et al. Sexual dysfunction in infertile men: a systematic review and meta-analysis. Sex Med. 2022;10:100528. 35636279 10.1016/j.esxm.2022.100528 PMC9386642

[11] Evgeni E, Sabbaghian M, Saleh R, et al. Sperm DNA fragmentation test: usefulness in assessing male fertility and assisted reproductive technology outcomes. Panminerva Med. 2023;65:135–47. 37103485 10.23736/S0031-0808.23.04836-X

[12] Rosen RC, Cappelleri JC, Smith MD, Lipsky J, Peña BM. Development and evaluation of an abridged, 5-item version of the International Index of Erectile Function (IIEF-5) as a diagnostic tool for erectile dysfunction. Int J Impot Res. 1999;11:319–26. 10637462 10.1038/sj.ijir.3900472

[13] Morley JE, Charlton E, Patrick P, et al. Validation of a screening questionnaire for androgen deficiency in aging males. Metabolism. 2000;49:1239–42. 11016912 10.1053/meta.2000.8625

[14] Whooley MA, Avins AL, Miranda J, Browner WS. Case-finding instruments for depression. Two questions are as good as many. J Gen Intern Med. 1997;12(7):439–45. 9229283 10.1046/j.1525-1497.1997.00076.x PMC1497134

[15] Cooper TG, Noonan E, von Eckardstein S, et al. World Health Organization reference values for human semen characteristics. Hum Reprod Update. 2010;16:231–45. 19934213 10.1093/humupd/dmp048

[16] Samplaski MK, Dimitromanolakis A, Lo KC, et al. The relationship between sperm viability and DNA fragmentation rates. Reprod Biol Endocrinol. 2015;13:42. 25971317 10.1186/s12958-015-0035-y PMC4432573

[17] Pyke RE. Sexual performance anxiety. Sex Med Rev. 2020;8:183–90. 31447414 10.1016/j.sxmr.2019.07.001

[18] Shiri R, Koskimäki J, Tammela TL, Häkkinen J, Auvinen A, Hakama M. Bidirectional relationship between depression and erectile dysfunction. J Urol. 2007;177:669–73. 17222655 10.1016/j.juro.2006.09.030

[19] Rosen RC, Riley A, Wagner G, Osterloh IH, Kirkpatrick J, Mishra A. The international index of erectile function (IIEF): a multidimensional scale for assessment of erectile dysfunction. Urology. 1997;49:822–30. 9187685 10.1016/s0090-4295(97)00238-0

[20] Yu X, Zhang X, Wang Q. Sexual dysfunction is more common among men who have high sperm DNA fragmentation or teratozoopermia. Sci Rep. 2022;12:22427. 36575203 10.1038/s41598-022-27006-z PMC9794705

[21] Yu X, Zhang S, Zhang XY, Wang Q. Sperm quality impairment in males of couples with pregnancy loss is correlated with sexual dysfunction: a cross-sectional study. Reprod Biol Endocrinol. 2023;21:11. 36709287 10.1186/s12958-023-01067-9 PMC9883875

[22] Meeker JD, Godfrey-Bailey L, Hauser R. Relationships between serum hormone levels and semen quality among men from an infertility clinic. J Androl. 2007;28:397–406. 17135633 10.2164/jandrol.106.001545

[23] Wang C, Swerdloff RS. Limitations of semen analysis as a test of male fertility and anticipated needs from newer tests. Fertil Steril. 2014;102:1502–7. 25458617 10.1016/j.fertnstert.2014.10.021 PMC4254491

[24] Wang Y, Jiang R. Androgens and erectile dysfunction: from androgen deficiency to treatment. Sex Med Rev. 2024;12:458–68. 38719619 10.1093/sxmrev/qeae030

[25] Cho CL, Esteves SC, Agarwal A. Novel insights into the pathophysiology of varicocele and its association with reactive oxygen species and sperm DNA fragmentation. Asian J Androl. 2016;18:186–93. 26732105 10.4103/1008-682X.170441 PMC4770484

[26] Song SH, Kim DS, Yoon TK, Hong JY, Shim SH. Sexual function and stress level of male partners of infertile couples during the fertile period. BJU Int. 2016;117:173–6. 26074135 10.1111/bju.13201

